# Long-term oncologic outcomes of transanal TME compared with transabdominal TME for rectal cancer: a systematic review and meta-analysis

**DOI:** 10.1007/s00464-021-08615-7

**Published:** 2021-06-24

**Authors:** Jae Young Moon, Min Ro Lee, Gi Won Ha

**Affiliations:** Research Institute of Clinical Medicine of Jeonbuk National University-Biomedical Research Institute of Jeonbuk National University Hospital, San 2-20 Geumam-dong, Deokjin-gu, Jeonju, Jeonbuk 561-180 South Korea

**Keywords:** Rectal cancer, Transanal TME, Transabdominal TME, Prognosis, Survival

## Abstract

**Background:**

Transanal total mesorectal excision (TaTME) appears to have favorable surgical and pathological outcomes. However, the evidence on survival outcomes remains unclear. We performed a meta-analysis to compare long-term oncologic outcomes of TaTME with transabdominal TME for rectal cancer.

**Methods:**

PubMed, EMBASE, and the Cochrane Library were searched. Data were pooled, and overall effect size was calculated using random-effects models. Outcome measures were overall survival (OS), disease-free survival (DFS), and local and distant recurrence.

**Results:**

We included 11 nonrandomized studies that examined 2,143 patients for the meta-analysis. There were no significant differences between the two groups in OS, DFS, and local and distant recurrence with a RR of 0.65 (95% CI 0.39–1.09, I^2^ = 0%), 0.79 (95% CI 0.57–1.10, I^2^ = 0%), 1.14 (95% CI 0.44–2.91, I^2^ = 66%), and 0.75 (95% CI 0.40–1.41, I^2^ = 0%), respectively.

**Conclusion:**

In terms of long-term oncologic outcomes, TaTME may be an alternative to transabdominal TME in patients with rectal cancer. Well-designed randomized trials are warranted to further verify these results.

Total mesorectal excision (TME) has been considered the standard surgical procedure for patients with rectal cancer since it was first described in 1982 by Heald [1]. This procedure was initially performed with an open abdominal approach, and laparoscopic TME has been recently suggested as an alternative to open TME [2–4]. However, the surgical technique is complex and requires extensive experience to safely perform for high-quality surgical resection and good oncologic outcomes, particularly in patients with lower rectal cancer. With recent advances in minimally invasive surgery, a transanal and laparoscopic combined approach was introduced as transanal TME (TaTME), and this was proposed as a possibility for overcoming the technical difficulties of transabdominal TME [5]. Although a majority of rectal cancers can be safely operated on with the transabdominal approach, difficult anatomical conditions, unfavorable tumor characteristics, or a combination of these factors can lead to difficulties. Narrow pelvis, fatty mesorectum, male sex, high BMI, and anterior-located large tumor are risk factors for noncurative resection [6]. The transanal approach may provide better access and visualization of the distal part of the rectum.

Many studies, including a meta-analysis, have reported favorable results in terms of perioperative, pathological, and functional outcomes in patients receiving TaTME for rectal cancer. The above-mentioned risk factors, in combination with the difficulty of perpendicular division of the rectum, seem to be related to circumferential resection margin (CRM) involvement, incompleteness of TME, and anastomotic leakage, which are considered to have negative oncologic impacts [7–13]. However, despite favorable results for CRM involvement, incompleteness of TME, and anastomotic leakage in TaTME, there is still a lack of evidence on long-term oncologic outcomes to support its widespread introduction. Therefore, our aim was to conduct a systematic review and meta-analysis to evaluate survival outcomes such as 2-year or 3-year survivals, or if possible 5-year survivals, and recurrence rates of TaTME in comparison with transabdominal TME in patients with rectal cancer. Evaluated outcomes were overall survival (OS), disease-free survival (DFS), and local and distant recurrence.

## Methods

This meta-analysis followed the recommendations of the Preferred Reporting Items for Systematic Reviews and Meta-Analyses (PRISMA) statement [14]. Multiple comprehensive databases were searched for studies that assessed the long-term oncologic outcomes of TaTME compared with transabdominal TME for rectal cancer. The study protocol used Cochrane Review Methods [15]. IRB approval was not needed for this article.

### Data and literature sources

Studies were identified from PubMed (January 1, 1976 to April 7, 2020), EMBASE (January 1, 1985 to April 7, 2020), and the Cochrane Central Register of Controlled Trials (CENTRAL) (January 1, 1987 to April 7, 2020). There were no restrictions regarding the year of publication, and articles in any language were permitted for review. The search terms were "rectal cancer," "transanal TME," "recurrence," "prognosis," and "survival." After the preliminary electronic search, further articles were searched for manually to retrieve additional studies. Finally, all articles were assessed individually for inclusion.

### Study selection and data extraction

Article titles and abstracts were screened and full texts were independently reviewed by two reviewers (JY Moon and GW Ha) according to the selection criteria. Any differences in judgment regarding inclusion were resolved through discussion between the reviewers.

The included studies assessed survival outcomes, including OS, DFS, local recurrence, and distant recurrence, in patients with rectal cancer who were treated with TaTME or transabdominal TME. All of the surgical modalities such as open, laparoscopic, and robotic surgery were included in both TME approaches if possible. Studies were excluded if they (i) did not compare TaTME with transabdominal TME; (ii) assessed patients with stage IV or recurred rectal cancer; (iii) assessed only patients who received abdominoperineal resection; (iv) had no extractable data and authors were unavailable to provide additional information; or (v) were case series with fewer than 10 patients.

All eligible studies were reviewed and all relevant data were extracted by the two reviewers independently using a data extraction form designed before the review. The variables recorded were (i) standard publication information, including year of publication, name of the first author, and number of patients; (ii) clinical and demographic characteristics of included studies; and (iii) outcomes (OS, DFS, local recurrence, and distant recurrence).

### Assessment of methodological quality

The methodological quality of the studies included in the meta-analysis was assessed using the Newcastle–Ottawa quality scale (NOS), which attributes a maximum of 9 points to each study and categorizes a study with a score of 6 or more as “high quality” [16]. The quality of the included studies was analyzed using 3 categories: patient selection, comparability, and outcome assessment.

### Statistical analysis

For dichotomous outcomes, relative risk (RR), variance, and 95% confidence interval (CI) were determined in the meta-analysis. The presence and amount of heterogeneity were assessed using the Q test and I^2^ index, respectively; a p-value less than 0.1 was considered statistically significant [17]. The DerSimonian-Laird random-effects model (REM) was used to pool data in light of cross-study heterogeneity [18].

First, we performed a meta-analysis to evaluate survival outcomes such as OS, DFS, and local and distant recurrence of TaTME in comparison with transabdominal TME in patients with rectal cancer. Second, we performed a meta-analysis to compare CRM involvement, incompleteness of TME, and anastomotic leakage between the two groups. Sensitivity analyses were performed to assess the robustness of the meta-analysis findings [19, 20]. First, studies with a higher rate of CRM involvement in the transabdominal TME group than in the TaTME group were analyzed. Second, studies with a higher rate of incomplete TME in the transabdominal TME group than in the TaTME group were analyzed. Third, studies with a higher rate of anastomotic leakage in the transabdominal TME group than in the TaTME group were analyzed. Fourth, studies with large outlying effects or studies with a score less than 6 in the NOS scale, indicating low quality, were excluded. Fifth, the trim-and-fill method and analysis with an alternative effect size were performed.

Funnel plots were used to determine the presence of publication bias by visual inspection of funnel plots and the Egger-weighted linear regression test; a p-value less than 0.1 was considered statistically significant [21, 22]. Data analyses were performed using Review Manager software (version 5.4) from the Cochrane Collaboration and Comprehensive Meta-Analysis software (version 3).

## Results

### Description of studies

**Fig. 1:**
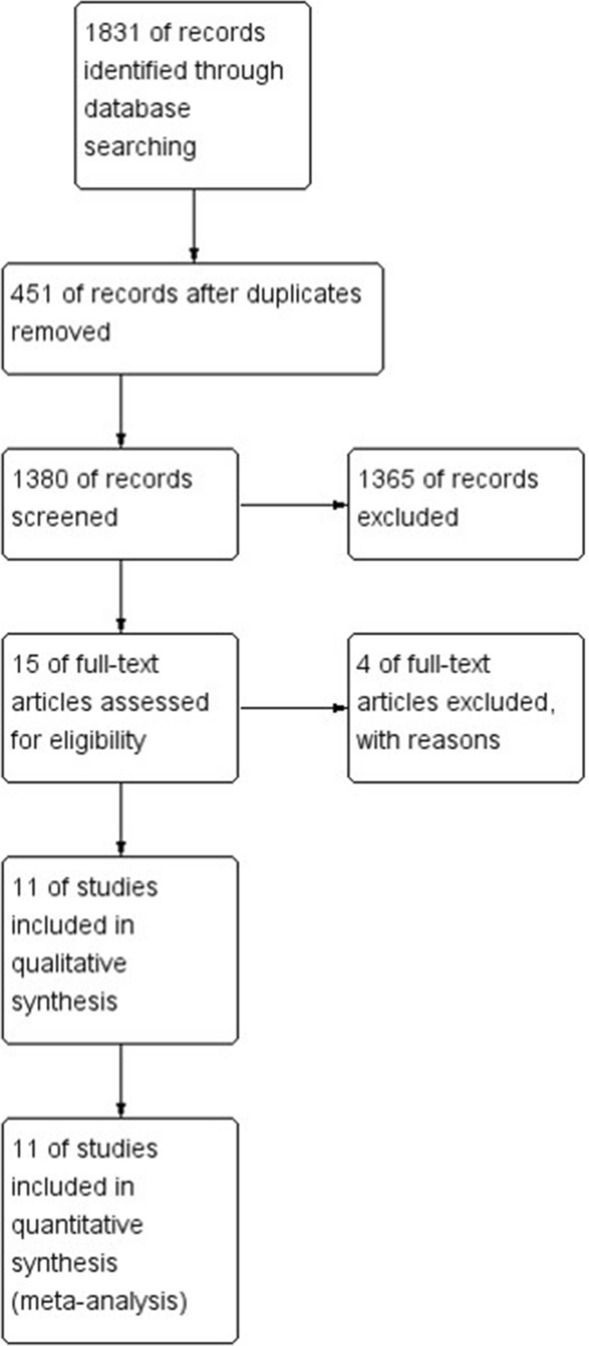
PRISMA flow diagram

The predefined search strategy identified 1,831 potentially relevant articles. We excluded 451 articles because they were duplicates and 1,365 articles because their titles and abstracts did not fulfill the selection criteria. After full text review of the remaining 15 articles, we excluded 4 articles because of the exclusion criteria of this study. Therefore, we included 11 nonrandomized studies that examined 2,143 patients for qualitative analysis and meta-analysis (Fig. 1). Among included patients, 529 patients received TaTME. Six studies evaluated OS and DFS [23–28], 11 studies evaluated local recurrence [23–33], and five studies evaluated distant recurrence [23, 26, 30–32]. Most of the included studies evaluated patients who underwent laparoscopic TaTME, while one study evaluated patients who underwent open TaTME [24]. Most of the included studies evaluated patients who underwent transabdominal TME with the laparoscopic approach only; two studies included patients who underwent transabdominal TME with laparoscopic or open approaches [24, 33], and one study included patients who underwent transabdominal TME with a robotic TME approach [30]. Evaluation of methodological quality showed that all studies scored at least 6 points (≥6) on the NOS scale. Tables 1 and 2 summarize the characteristics of the included studies.

**Table 1:** Summary of the included studies

Study	Design	Country	Period	Number		Age		Gender (M/F), n		BMI (kg/m2)		ASA score		Inclusion criteria	Exclusion criteria	Surgical method	Follow up (months)	Oncologic outcomes	NOS
				TaTME	TME	TaTME	TME	TaTME	TME	TaTME	TME	TaTME	TME						
De'Angelis (2015) [22]	Retro	France	2011–2014	32	32	64.91^a^	67.16^a^	21/11	21/11	25.19^a^	24.53^a^	I + II:96.9% III + IV:3.1%	I + II:96.9% III + IV:3.1%	Up to 5 cm from the AV	NR	TaTME, Lap TME	32.06/ 62.91^a^	LR, DR, 2-yr OS, DFS	7
Marks (2016) [28]	Retro	USA	2012–2014	17	17	59^a^	60^a^	NR		26.4^a^	25.9^a^	NR		Tumors in the distal 4 cm rectum to the ARR	NR	TaTME, Lap TME	19.5/ 42.3^a^	LR	6
Lelong (2017) [24]	Retro	France	2008–2013	34	38	NR		23/11	22/16	24 (18.6–45)^b^	24.2 (17.7–32.7)^b^	I:17.6% II:70.6% III:11.8%	I:23.7% II:71% III:5.3%	Some resectable mets were included	T4 tumors, nonresectable mets, peritoneal carcinosis	TaTME, Lap TME	31.9 (29.3–42) / 53.3 (8–95)^b^	LR, 2-yr OS, DFS	7
Xu (2017) [23]	Retro	China	2006–2015	74	41	59 ± 12.6^a^	62.4 ± 11.2^a^	115/0		25 ± 2.8	24.8 ± 2.3	I:10.8% II:58.1% III:31.1%	I:14.6% II:58.5% III:26.8%	Tumor ≤ 5 cm from the AV, no distant mets, tumor volume ≥ 4 cm	Tumor invasion in the external sphincter, pelvic floor muscles	TaTME*, Lap or open TME	46.1 ± 25.6^a^	LR, 5-yr OS, DFS	8
Denost (2018) [25]	Pros	France	2008–2012	50	50	64 (39–82)^b^	63 (31–90)^b^	37/13	32/18	25.1 (17.3–33.2-)^b^	25.6 (18.3–38.3)^b^	I:68% II:30:% III:2%	I:60% II:38% III:2%	Low rectal cancer suitable for sphincter-preserving surgery with hand-sewn coloanal anastomosis	High and mid rectal cancer, stapled anastomosis, APR, open surgery, local excision	TaTME, Lap TME	61.3 (2–88.2) / 55.4 (1–92.2)^b^	LR, DR, 5-yr OS, DFS	9
Lee (2018) [29]	Retro	Korea	2013–2014	21	24	< 60: 10/18 ≥ 60: 11/6**		16/5	13/11	24.4 ± 3.44^a^	23.6 ± 3.0^a^	I:38.1% II:57.1% III:4.8%	I:29.2% II:66.7% III:4.1%	Rectal adenocarcinoma, restorative proctectomy	Stage IV	TaTME, Robotic TME	20.1/22.0^b^	LR, DR	6
Mege (2018) [30]	Retro	France	2015–2017	34	34	58 ± 14^a^	59 ± 13^a^	23/11	23/11	25 ± 4^a^	25 ± 3^a^	I:12% II:85% III:3%	I:27% II:68% III:6%	Lower rectal cancer	Mid or high rectal cancer, APR	TaTME, Lap TME	13 ± 6/ 25 ± 14^a^	LR, DR	7
Chen P (2019) [27]	Retro	Taiwan	2013–2015	50	100	57.3 ± 11.9^a^	58.3 ± 11.3^a^	38/12	76/24	24.2 ± 3.7^a^	24.6 ± 3.1^a^	I/II:66% III:34%	I/II:69% III:31%	Stage II–III, Mid or lower rectal adenocarcinoma, received nCRT	Stage IV	TaTME, Lap TME	44.3 ± 10.5/ 84.5 ± 41.6^a^	LR, 3-yr OS, DFS	7
Chen YT (2019) [26]	Retro	Taiwan	2008–2018	39	64	62 ± 14.9^a^	64 ± 12.2^a^	29/10	42/22	25.4 ± 4^a^	24.6 ± 3.3^a^	I:12.8% II:71.8% III:15.4%	I:7.8% II:82.8% III:9.4%	Rectal adenocarcinoma 7 cm from the AV, stage I–III	Cancer perforation, T4, Stage IV, APR	TaTME, Lap TME	17.5 ± 8.8/ 37.5 ± 23.7^a^	LR, 2-yr DFS, OS	6
Gordeyev (2019) [31]	Retro	Russia	2013–2017	26	26	56.5 (25–68)^b^	63 (38–78)^b^	26/0	26/0	28.3 (25.4–36.4)^b^	29.2 (25.2–35.1)^b^	NR		Rectal cancer cT1-4aN0-2M0, combination of male gender, BMI(≥ 25 mg/m2), CRT	Synchronous or metachronous tumors, ECOG > 1, partial TME	TaTME, Lap TME	28.2^b^	LR, DR	6
Wasmuth (2020) [32]	Pros	Norway	2014–2018	152	1188	NR		109/48	NR	NR		NR		Rectal cancer	Stage IV	TaTME, Lap or open TME	19.5 (0–51) ^b^	LR	6

*Retro* Retrospective observational study, *Pros* Prospective observational study, *TaTME* Transanal total mesorectal excision, *ASA* American society of anesthesiologists, *ARR* anorectal ring, *AV* anal verge, *nCRT* neoadjuvant chemoradiotherapy, *TATA* Transanal abdominal transanal, *NOS* Newcastle–Ottawa scale, *NR* not reported

^a^Mean

^b^Median

^*^TaTME was performed in an open fashion

^**^Number of patients

**Table 2:** Clinical characteristics of the included studies

Study	Pathological Stage		Tumor location from AV (cm)		nCRT received (%)		RT, cGy	Concurrent Chemo agent	Interval to surgery	CRM positive, mean CRM (mm)		DRM positive, mean DRM (mm)		Incompleteness of TME		LN harvest, n		Anastomosis type, Anastomotic leaks		Mortality	AdjCtx	Recurrence	Survival rate
	TaTME	TME	TaTME	TME	TaTME	TME				TaTME	TME	TaTME	TME	TaTME	TME	TaTME	TME	TaTME	TME				
De'Angelis (2015) [22]	CR:12.5% T1:9.4% T2:37.5% T3:34.4% T4:6.2% N0:84.4% N1:15.6%	CR:18.8% T1:6.2% T2:28.1% T3:40.6% T4:6.2% N0:78.1% N1:18.8% N2:3.1%	4 (2.5–5)^b^	3.7 (2.5–5) ^b^	84.4	71.9	4500–5040	5FU	6–8 weeks	3.1%, 9.68	9.4%, 9.19	6.2%, 21.32	0%, 22.92	Complete: 84.4% Nearly complete: 9.4% Incomplete: 6.2%	Complete: 75% Nearly complete: 12.5% Incomplete: 12.5%	17^a^	19^a^	Hand-sewn, AL 12.5%	Hand-sewn, AL 21.9%	0%	NR	LR 3.1% vs 6.3%, DR 3.1% vs 6.3%	OS 95.5% vs 96.6%, DFS 90.5% vs 85.2%
Marks (2016) [28]	uT2:29.4% uT3:70.6%	uT2:23.5% uT3:76.5%	0.9 (-2.0–3.0)*	0.8 (-1.5–4.0)*	100		5326/ 5412^a^	5FU/ Xeloda	NR	0%, NR	5.9%, NR	0%, NR	0%, NR	Complete: 88.2% Nearly complete: 11.8%	Complete: 88.2% Nearly complete: 5.9% Incomplete: 5.9%	7.5^a^	8.5^a^	Hand-sewn, AL 0%	NR	NR	NR	LR 5.9% vs 0%	NR
Lelong (2017) [24]	CR:20.6% T1:18.8% T2:26.5% T3:44.1% N0:73.5% N1:20.6% N2:5.9%	CR:31.6% T1:13.2% T2:26.3% T3:26.3% T4:2.6% N0:86.8% N1:13.2%	NR		88.2	92.1	4500–5000	Xeloda	NR	5.9%, 1–2: 20.6% > 2: 73.5%	10.5%, 1–2: 7.9% > 2: 81.6%	0%, NR	2.6%, NR	Complete: 55.9% Nearly complete: 44.1%	Complete: 52.6% Nearly complete: 42.1% Incomplete: 5.3%	14 (6–34) ^b^	12 (4–25) ^b^	Hand-sewn, AL 5.9%	Hand-sewn, AL 15.8%	NR	NR	LR 5.7% vs 5.3%	OS 100% vs 95%, DFS 86% vs 88%
Xu (2017) [23]	T1:5.4% T2:40.5% T3:54.1% I:37.8% II:39.2% III:23%	T1:4.9% T2:29.3% T3:65.9% I:29.3% II:31.7% III:39%	4 (1–5) ^b^	4 (0.5–5) ^b^	35.6	12.5	4500–5000	Xeloda	6–8 weeks	2.7%, NR	4.9%, NR	0%, 17.9 ± 4.9^a^	0%, 16.9 ± 5.3^a^	Complete: 90.5% Nearly complete: 9.5%	Complete: 70.7% Nearly complete: 22% Incomplete: 7.3%	NR		Hand-sewn, AL 2.7%	Hand-sewn, AL 4.9%	0%	64% vs 55.2%	LR 5.4% vs 14.6%	OS 81% vs 75.5%, DFS 79.5% vs 61.5%
Denost (2018) [25]	T0–2:60% T3–4:40% N0:66% N1–2:34%	T0–2:56% T3–4:44% N0:58% N1–2:42%	4 (2–6)^b^	4 (2–6)^b^	80	88	4500	5FU, Xeloda	6 weeks	4%, 7 (0–20)^b^	18%, 5 (0–20)^b^	2%, 10 (1–30)^b^	8%, 10 (0–30)^b^	Complete: 70% Nearly complete: 18% Incomplete: 12%	Complete: 62% Nearly complete: 26% Incomplete: 12%	17 (2–30)^b^	17 (9–40)^b^	Hand-sewn, AL 2%	Hand-sewn, AL 10%	0% vs 2%	24% vs 38%	LR 2.6% vs 4.8%, DR 12% vs 20%	OS 87% vs 74.4%, DFS 73.9% vs 71.9%
Lee (2018) [29]	T0:19% T1:19% T2:19% T3:38.1% T4:4.8% N0:71.4% N1:23.8% N2:4.8% 0:23.8% I:23.8% II:23.8% III:28.6%	T0:8.3% Tis:8.3% T1:16.7% T2:37.5% T3:29.2% N0:87.5% N1:12.5% 0:16.7% I:50% II:20.8% III:12.5%	6.1 ± 1.63	5.2 ± 1.99	66.7	50	NR	NR	NR	NR, > 10: 66.7% 5–10: 23.8% 1–5: 4.8% ≤ 1: 4.8%	NR, > 10: 70.8% 5–10: 12.5% 1–5: 8.3 ≤ 1: 8.3%	NR, 22 ± 12.8^a^	NR, 19 ± 10.6^a^	Complete: 90.5% Nearly complete: 9.5%	Complete: 100%	NR		Stapled 85.7%, Hand-sewn 14.3% AL 4.8%	Stapled 62.5%, Hand-sewn 37.5% AL 12.5%	0%	NR	LR 4.8% vs 0%, DR 9.5% vs 4.2%	NR
Mege (2018) [30]	CR:29% Tis:3% T1:3% T2:24% T3:38% T4:3% N + :44% M + :9% I:21% II:3% III:38% IV:9%	CR:15% Tis:6% T1:12% T2:32% T3:32% T4:3% N + :26% M + :9% I:47% II:9% III:21% IV:9%	1.3 ± 1.1*	2.2 ± 1.7*	85	85	5000	NR	10 weeks	12%, < 1: 12%	15% < 1: 6% 1: 9%	3%, 13 ± 9^a^	3%, 14 ± 12^a^	Complete: 53% Nearly complete: 27% Incomplete: 21%	Complete: 79% Nearly complete: 9% Incomplete: 12%	14 ± 10^a^	14 ± 8^a^	Hand-sewn, AL 12%	Hand-sewn, AL 15%	0%	50% vs 32%	LR 0% vs 0%, DR 15% vs 18%	NR
Chen P (2019) [27]	CR:16% I:26% II:24% III:34%	CR:17% I:20% II:33% III:30%	5.8 ± 2.1^a^	6.7 ± 2.0^a^	100	100	5040	Xeloda	6–10 weeks	4%, 11.8 ± 7.5^a^	10%, 11.1 ± 7.7^a^	NR, 2.4 ± 1.2^a^	NR, 1.5 ± 0.9^a^	NR		16.7 ± 7.8^a^	17.4 ± 8.9^a^	Stapled 68%, Hand-sewn 32% AL 14%	Stapled 67%, Hand-sewn 33% AL 9%	NR	NR	LR 7.5% vs 8.5%	OS 98% vs 99%, DFS 82% vs 82%
Chen YT (2019) [26]	CR:10.3% I:41% II:17.9% III:30.8%	CR:6.3% I:31.3% II:20.3% III:42.1%	4.3 ± 1.4^a^	5.8 ± 1.2^a^	39	48	NR	NR	NR	NR, < 1: 0% ≥ 1: 100%	NR, < 1: 7.8% ≥ 1: 92.2%	NR, 16 ± 14^a^	NR, 19 ± 13^a^	NR		20.8 ± 9^a^	18.8 ± 8.1^a^	Stapled 89.7%, Hand-sewn 10.3% AL 2.6%	Stapled 100% AL 0%	0%	NR	LR 0% vs 4.7%	DFS 90% vs 91%, OS 97% vs 89%
Gordeyev (2019) [31]	T0:23.1% T1–2:26.9% T3:46.2% T4a:3.8% N + :50%	T0:19.2% T1–2:23% T3:53.9% T4a:3.8% N + :38.5%	7 (4–9)^b^	7 (4–11)^b^	100	100	NR	NR	NR	7.7%, NR	11.5%, NR	NR, 30 (7–60)^b^	NR, 25 (9–70)^b^	Complete/Nearly complete: 84.6% Incomplete: 15.4%	Complete/Nearly complete: 84.6% Incomplete: 15.4%	12 (5–60)^b^	16 (2–54)^b^	Stapled 84%, Hand-sewn 16% AL 11.5%	Stapled 68%, Hand-sewn 32% AL 11.5%	0%vs3.8%	NR	LR 3.8% vs 0%, DR 3.8% vs 3.8%	NR
Wasmuth (2020) [32]	T0:5.1% T1:17.2% T2:36.3% T3:36.3% T4:5.1% N0:68.8% N1:18.5% N2:12.7%	T0:6% T1:8.3% T2:33.1% T3:48.7% T4:3.7% N0:66.5% N1:23.4% N2:10%	8 (2–13)^b^	NR	21	39	NR	NR	NR	5.1%, NR	NR	7.6%, NR	NR	NR		NR		NR, AL 8.4%	NR, AL 4.5%	3.2% vs 1.3%	NR	LR 11.6% vs 2.4%	NR

*nCRT* neoadjuvant chemoradiotherapy, *Mortality* 30 days mortality, *Adj Ctx* adjuvant chemotherapy

^*^Mean distant from the anorectal ring

^a^Mean

^b^Median

### Long-term oncologic outcomes of TaTME compared with transabdominal TME

**Fig. 2:**
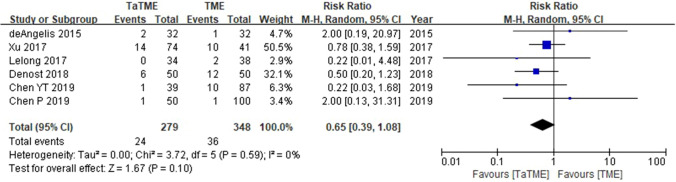
Forest plot of data on OS in patients with rectal cancer (TaTME vs. transabdominal TME)

**Fig. 3:**
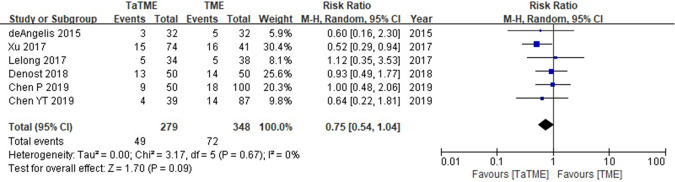
Forest plot of data on DFS in patients with rectal cancer (TaTME vs. transabdominal TME)

**Fig. 4:**
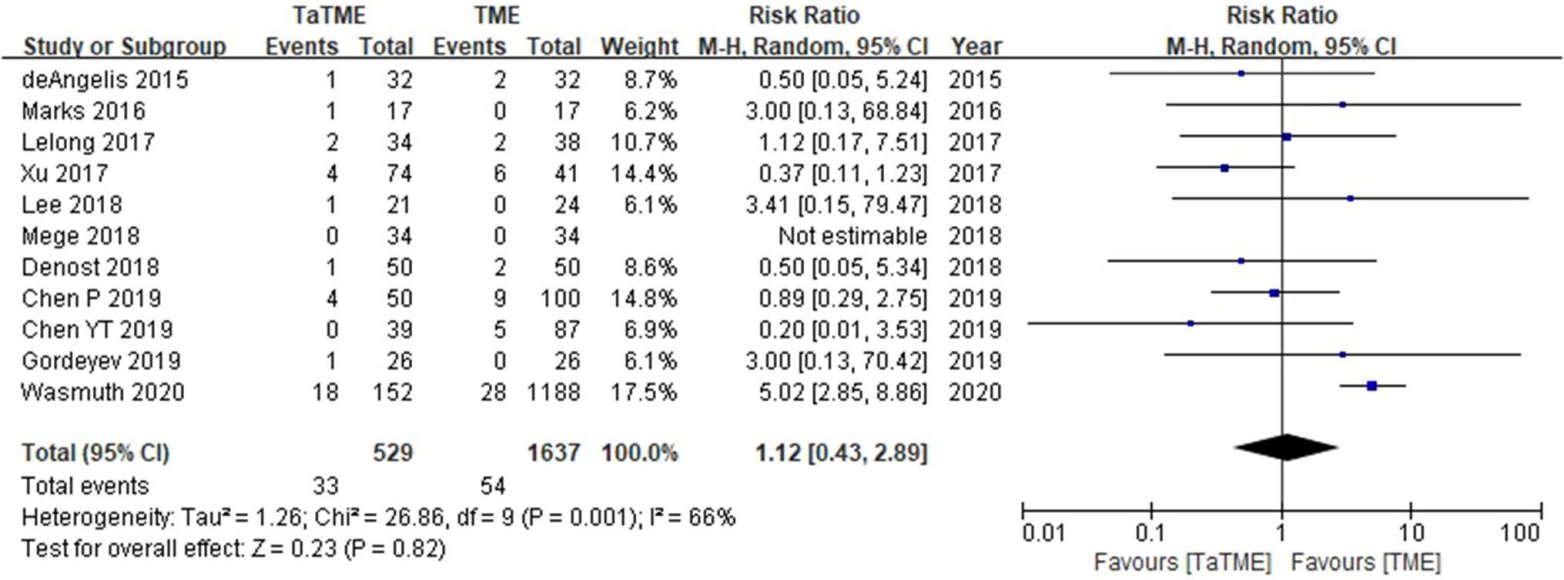
Forest plot of data on local recurrence in patients with rectal cancer (TaTME vs. transabdominal TME)

**Fig. 5:**
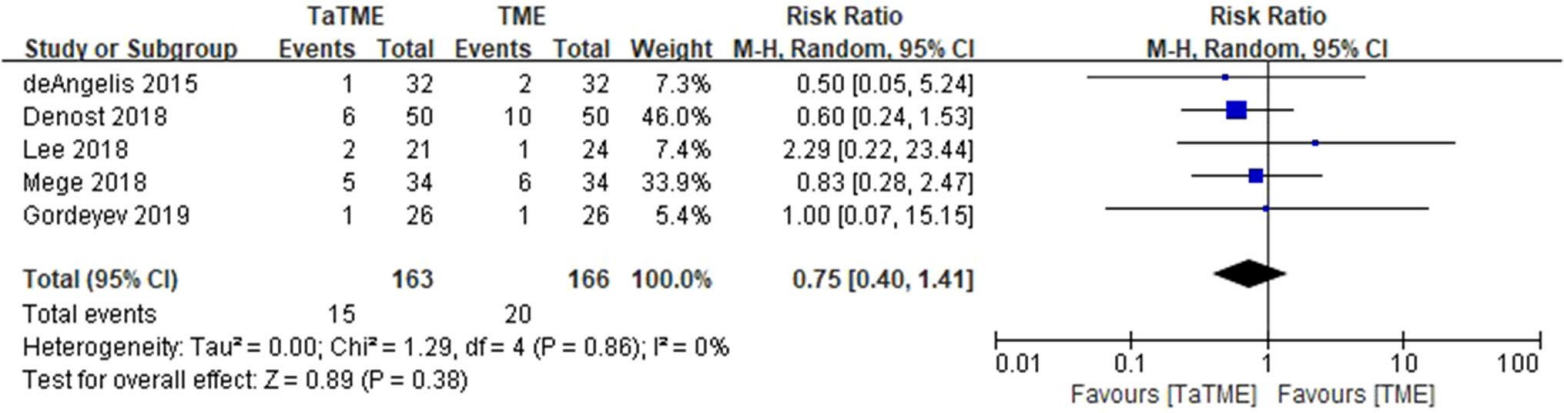
Forest plot of data on distant recurrence in patients with rectal cancer (TaTME vs. transabdominal TME)

Analysis of oncologic outcomes for TaTME in patients with rectal cancer indicated that 6 studies (604 patients) reported data on OS; there were no significant survival differences between TaTME and transabdominal TME (risk ratio [RR] = 0.65, 95% confidence interval [CI] = 0.39–1.09, I^2^ = 0%) (Fig. 2). Six studies (604 patients) reported data on DFS; there were no significant survival differences between the two groups (RR = 0.79, 95% CI = 0.57–1.10, I^2^ = 0%) (Fig. 3). Eleven studies (2,143 patients) reported data on local recurrence; there were no significant differences between two groups (RR = 1.14, 95% CI = 0.44–2.91, I^2^ = 66%) (Fig. 4). Five studies (329 patients) reported data on distant recurrence; there were no significant differences between two groups (RR = 0.75, 95% CI = 0.40–1.41, I^2^ = 0%) (Fig. 5). Sensitivity analyses using predefined methods indicated that the results of these meta-analyses were robust.

### Analyses of CRM involvement, incompleteness of TME, and anastomotic leakage

**Fig. 6:**
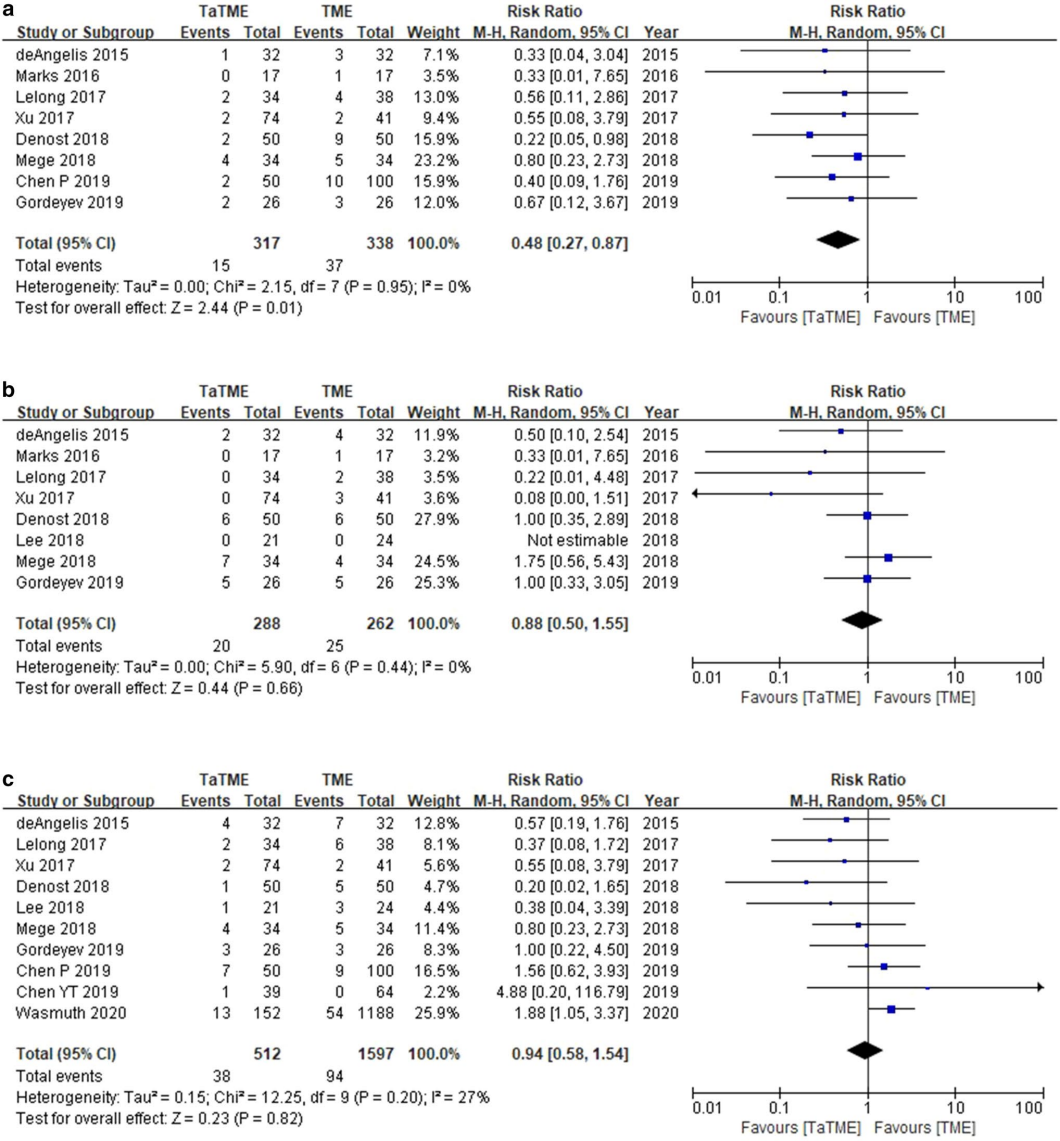
**a** Analysis of CRM involvement, **b** analysis of incompleteness of TME, and **c** analysis of anastomotic leakage (TaTME vs. transabdominal TME)

Comparing CRM involvement between the two groups, the TaTME group was associated with better outcomes, with a RR of 0.44 (95% CI 0.27–0.87, I^2^ = 0%) (Fig. 6a). Analysis to compare incompleteness of TME showed no significant differences between TaTME and transabdominal TME groups, with a RR of 0.88 (95% CI 0.50–1.55, I^2^ = 0%) (Fig. 6b). Analysis to compare anastomotic leakage also showed no significant differences between TaTME and transabdominal TME groups, with a RR of 0.94 (95% CI 0.58–1.54, I^2^ = 27%) (Fig. 6c).

### Analysis of oncologic outcomes according to rate of CRM involvement

**Fig. 7:**
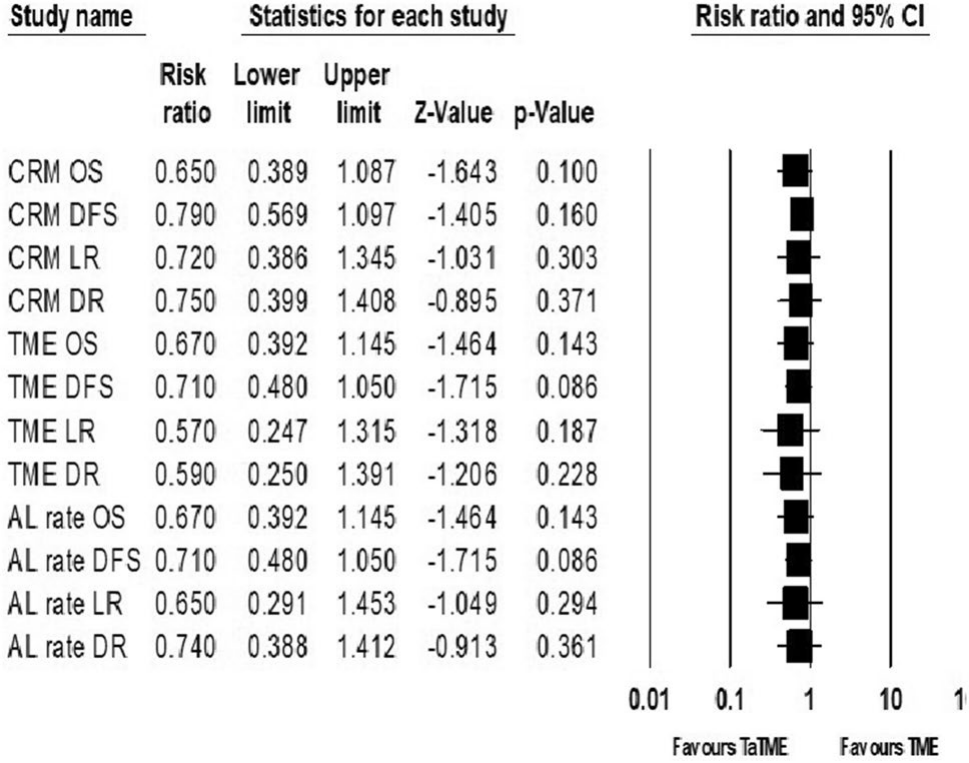
Sensitivity analysis of long-term oncologic outcomes related to CRM involvement, incompleteness of TME, and anastomotic leakage

Analysis of studies with a higher rate of CRM involvement in the transabdominal TME group than in the TaTME group showed no significant differences between the two groups in analysis of OS, DFS, local recurrence, and distant recurrence with a RR of 0.65 (95% CI 0.39–1.09, I^2^ = 0%), 0.79 (95% CI 0.57–1.10, I^2^ = 0%), 0.72 (95% CI 0.39–1.36, I^2^ = 0%), and 0.75 (95% CI 0.40–1.41, I^2^ = 0%), respectively (Fig. 7).

### Analysis of oncologic outcomes according to rate of TME incompleteness

Analysis of studies with a higher rate of incomplete TME in the transabdominal TME group than in the TaTME group showed no significant differences between the two groups in analysis of OS, DFS, local recurrence, and distant recurrence with a RR of 0.67 (95% CI 0.39–1.14, I^2^ = 0%), 0.71 (95% CI 0.48–1.05, I^2^ = 0%), 0.57 (95% CI 0.25–1.33, I^2^ = 0%), and 0.59 (95% CI 0.25–1.39, I^2^ = 0%), respectively (Fig. 7).

### Analysis of oncologic outcomes according to rate of anastomotic leakage

Analysis of studies with a higher rate of anastomotic leakage in the transabdominal TME group than in the TaTME group showed no significant differences between the two groups in analysis of OS, DFS, local recurrence, and distant recurrence with a RR of 0.67 (95% CI 0.39–1.14, I^2^ = 0%), 0.71 (95% CI 0.48–1.05, I^2^ = 0%), 0.65 (95% CI 0.29–1.45, I^2^ = 0%), and 0.74 (95% CI 0.39–1.42, I^2^ = 0%), respectively (Fig. 7).

### Publication bias

Publication bias was determined by visual inspection of funnel plots and the Egger-weighted linear regression test to assess any asymmetry in the funnel plots. The results showed that the funnel plots for local recurrence (*p* = 0.045) were asymmetrical, indicating a presence of publication bias.

## Discussion

To our knowledge, despite a relatively small number of included patients, this study is the first meta-analysis to compare long-term oncologic outcomes between TaTME and transabdominal TME. Since TaTME was introduced in 2010 [5], many studies have reported favorable perioperative, pathological, and functional outcomes, although little is known about the long-term oncologic outcomes of TaTME such as OS, DFS, and distant recurrence. Our findings on the long-term oncologic outcomes of TaTME may illustrate its oncologic safety and support its introduction and application.

Our meta-analysis showed no significant difference between TaTME and transabdominal TME in OS, DFS, local recurrence, and distant recurrence. The TaTME group had favorable CRM involvement compared with the transabdominal TME group. However, despite tendencies for lower rates of incompleteness of TME and anastomotic leakage in the TaTME group, there was no significant difference between the two groups in terms of incompleteness of TME and anastomotic leakage. Based on previous meta-analyses [11, 13], we considered lower rates of CRM involvement, incompleteness of TME, and anastomotic leakage in the TaTME group could demonstrate adequately performed TaTME procedures, which might show survival outcomes properly after overcoming the initial learning curve. Thus, we performed sensitivity analyses using predefined methods, such as analyses of long-term oncologic outcomes related to CRM involvement, incompleteness of TME, and anastomotic leakage, which indicated no statistical significance, suggesting the robustness of these results.

Studies have shown that CRM is an accepted surrogate marker for local recurrence and those with involved CRM have an increased risk of local recurrence [34, 35]. However, in our study, although the TaTME group had favorable CRM involvement and most included studies reported a higher rate of CRM involvement in the transabdominal TME group [23–32], margin involvement does not translate into significant differences in the rates of OS, DFS, distant recurrence, and local recurrence between the two groups. Another surrogate marker for local recurrence is the quality of the mesorectum [36]. In our study, analysis of incompleteness of TME showed no significance, and analysis of studies that reported a higher rate of incomplete mesorectum in the transabdominal TME group [23–26, 29] showed no significance in the rates of OS, DFS, distant recurrence, and local recurrence between the two groups. Anastomotic leakage may also have a negative effect on recurrence and survival outcomes [37–39]. In our study, analysis of anastomotic leakage showed no significance, and analysis of studies that reported a higher rate of anastomotic leakage in the transabdominal TME group [23–26, 30, 31] showed no significance in the rates of OS, DFS, distant recurrence, and local recurrence between the two groups. However, it is important to point out the relatively small number of included patients and the trends for better survival outcomes in TaTME group. The transanal approach with advances in technique and quality control will provide more patient data for analysis of the oncologic impact of TaTME. Consequently, as patient data increases, less CRM involvement, less TME incompleteness, and less anastomotic leakage may have a significantly positive effect on TaTME survival outcomes and recurrence.

Recently, TaTME for rectal cancer was suspended in Norway due to an unexpected higher recurrence rate after TaTME [40]. In our meta-analysis, except for one study [33], all included studies reported an acceptable local recurrence rate. After excluding this study, the result of local recurrence analysis had a trend for better outcomes in the TaTME group. One explanation may involve the technical aspect of rectal transection and air flow during dissection from the perineum, which could potentially allow the spread of tumor cells into the pelvic cavity [41]. Therefore, to ensure complete occlusion of the rectal lumen and reduce the possibility of tumor cells spreading, a modification of the technique to reinforce the purse-string has been proposed [42]. Before full-thickness incision of the rectum, placing a gauze swab in the lumen can also prevent tumor cell spillage [26].

There are some limitations to this study that make it difficult to draw strong conclusions. One limitation of this study is it lacks large randomized trials, and that the majority of the studies are retrospective and have a small number of patients. Second, there may be a potential heterogeneity among the included studies, even though we performed a sensitivity analysis. Clinical characteristics of patients may be various because comparative studies without randomization were included. Moreover, the procedures were performed by many different surgeons, and any non-standardized techniques used may have influenced the oncologic outcomes. Although TaTME is usually recommended as dissection of the distal one-third of the mesorectum [43], the level of rectal dissection via TaTME may vary between patients. Third, there are variations in the follow-up period among the included studies, and this might have affected the results.

In conclusion, although it remains in a stage of development, TaTME may offer favorable long-term oncologic outcomes and be an alternative to transabdominal TME in patients with distal rectal cancer. Well-designed large randomized trials are warranted to provide more definitive survival results.
